# Association of Non-Carious Cervical Lesions with Oral Hygiene Habits and Dynamic Occlusal Parameters

**DOI:** 10.3390/diagnostics9020043

**Published:** 2019-04-12

**Authors:** Satheesh B. Haralur, Abdulrahman Saad Alqahtani, Mohammed Shaya AlMazni, Mohammad Khalid Alqahtani

**Affiliations:** Department of Prosthodontics, College of Dentistry, King Khalid University, 62529 Abha, Saudi Arabia; Asz98850@gmail.com (A.S.A.); Alqady5050@gmail.com (M.S.A.); almazni55@gmail.com (M.K.A.)

**Keywords:** non-carious cervical lesions, occlusion and disocclusion time, abfraction, oral hygiene habits

## Abstract

Dentists routinely encounter non-carious cervical lesions (NCCLs) in daily clinical practices. The dental literature is contradictory regarding the occlusal etiology of NCCLs. NCCL is defined as the loss of dental structure at the cemento–enamel junction, with no association of bacteria. The aim of this study was to evaluate the association of dynamic occlusal factors and dental hygiene practices with NCCLs. In total, 100 participants were selected by the random stratified sampling method, 50 each for control and NCCL groups. Information regarding oral hygiene practices, including frequency, duration, force, and technique of brushing, was recorded. Dynamic occlusal parameters like initial contact, occlusion time (OT), disocclusion time (DT), and center of force were recorded with T-scan analysis. The obtained data were analyzed with Pearson’s correlation and binary logistic regression. We found that 68% of participants in the NCCL group and 31% in the control group utilized the horizontal brushing technique; 46% of NCCL group participants used hard brush against 7% of participants in the control group. The mean OT was 0.727 and 0.516 s for NCCL and control groups, respectively. The OT and left and right lateral DT were strongly related to NCCL, with *r*-values of 0.661, 0.642, and 0.534, respectively, with *p* ≤ 0.001. Using a hard toothbrush was found to be associated with NCCL. The NCCL group had extended mean occlusion time and disocclusion time in all eccentric mandibular movements.

## 1. Introduction

Tooth structure loss at the cemento–enamel junction (CEJ), with no involvement of bacteria, is referred to as non-carious cervical lesions (NCCLs) [1]. Clinically, NCCLs present as shallow or deep depressions, disc- or wedge-shaped defects at the CEJ. The clinicians encounter NCCLs routinely, and previous studies report large variation in their prevalence in different populations [2]. Borcic et al. [3] reported 13% of dentists encountered cervical C-shaped lesion. Conversely, Bergstrom et al. [4] found 85% of patients had a various degree of cervical tooth loss. Meanwhile, Hand et al. [5] reported an overall prevalence of NCCLs corresponding to 56 %, and Oginni et al. [6] found 62.3% of their studied Nigerian population had a cervical lesion. The large variation in prevalence is attributed to differences in the target populations, age, and diagnosis methods adopted by the researchers.

NCCLs often lead to the loss of structural integrity, tooth sensitivity, pulpal disorders, and compromised aesthetics [7]. Researches indicate it is the second most common cause of dental restoration of permanent teeth other than dental caries [8]. Researchers still disagree on the causative factors of NCCLs, though most of them consider them due to a multifactorial patho-dynamic mechanism [9]. The prevalence of NCCLs is expected to increase, as a result of the longer maintenance of teeth and longer life expectancy worldwide. Understanding the etiology of initiation and progression of NCCLs could help dentists to better manage these conditions. The clinical recognized mechanisms leading to NCCLs are abrasion, erosion, and abfraction. Tooth wear results from the interactive outcome of these three processes. Tooth structure loss is a complex mechanism that includes variables such as the properties of two opposing surfaces along with those of interfacial media like saliva and food. The enamel or restoration wear exceeding a physiological tooth wear may cause a destabilized occlusion. Hence, researchers are concerned about the abrasiveness and wear of restorative materials. Excessive wear of the restorative materials leads to the loss of organic matrix, and exposure of the inorganic component. The worn, rough surface encourage plaque accumulation and attrition with the opposing tooth. Matrix structure and fillers type and size determine the wear resistance of restorative materials [10,11]. Zafar et al. [12] reported that wear increases the surface hardness and elastic moduli of metallic restorations. Erosion is due predominantly to A chemical process, ascribed to the consumption of acidic food or drinks and acid reflux disorders [13]. Abrasion is mechanical wear due to frequent contact with objects or substances. The various factors described to influence abrasion are the brushing technique, force, and frequency, toothpaste abrasiveness, coarse foods, and harmful oral habits [14]. The abfraction process starts from the flexion of the tooth structure at the cervical region due to harmful occlusal forces. Researchers hypothesize that the occlusal factors nucleate and propagate the cracks at the CEJ, leading to NCCLs. The results of previous studies are inconclusive and contradictory regarding the association of abfraction with occlusal factors. Rees et al. [15] suggested bruxism is the main cause of wedge-shaped lesions at the CEJ. Lee et al. [16] postulated that tensile stress form masticatory force and malocclusion is the primary reason for angled notches at cervical lesions. Hammadeh et al. [17] investigated the effect of repetitive occlusal loading over cervical restoration and concluded the occlusal loading resulted in stress under the buccal cervical area, crack initiation, and loss of restoration. Conversely, Litonjua et al. [18] reported no evidence of enamel and dentin fracture in the cervical area due to axial and non-axial occlusal load.

The occlusal factors credited for eccentric loading are premature contacts, para-functional jaw movements, deglutition, and clenching [19]. Occlusal loading during mastication occurs in two stages. The initial phase is the contact of food with the occluding teeth, and the second phase involves the direct contact between opposing teeth. Though tooth–tooth contact has a short duration, it is predicted to be the chief etiological factor producing pathological occlusal stress [20]. The occlusal contact over the cuspal inclines of the posterior teeth could lead to cervical strain and contributes to NCCLs. Hence, the dynamic occlusal contacts during mastication can provide accurate details of the resultant forces. Digital occlusal analysis can examine the dynamic occlusal parameters such as occlusion time (OT), disocclusion time (DT) and sequence of occlusal contacts. The majority of the previous researches tested the association between occlusal factors and NCCLs in vitro, by finite-element analysis [15,21,22], and photo-elasticity studies [23]. The aim of this study was to assess the potential correlation between oral hygiene practices, occlusal parameters, and NCCLs. The null hypothesis tested was that there is no association between NCCL manifestations and various occlusal factors and different oral hygiene practices.

## 2. Materials and Methods

The method adopted in the study was descriptive, correlation research. Quantitative correlation study was selected to test the relationship between NCCLs and dynamic occlusal parameters and oral hygiene habits. On the basis of a medium effect size of 0.5, a power of 80%, and a significance level of 5% (*p* < 0.05), the sample size was determined to be 51 subjects per group and was rounded to 50 per group. The sample size was calculated with the G*Power software (version 3.1; University of Dusseldorf) [24]. In total, 100 participants, 50 each for control and NCCL, were selected by the random stratified sampling method among the patients visiting King Khalid University (KKU) dental clinics for treatment. The inclusion criteria were: age ≥19 years, teeth in occlusion, normal salivary function, and not under medication with analgesics, tranquilizers, psychiatric drugs. The research was a single-center study, conducted at dental clinics, King Khalid University, during the second semesters of 2017–2018. All participants received a thorough explanation regarding the study procedure, then gave their written consent before inclusion in the study. Clinical examination by the examiners was first optimized by a training exercise, followed by thorough explanation of the recording procedures. The investigators examined 10 participants (5 control and 5 NCCL), which were not included in the final analysis of the results. The procedure was repeated two times to determine intra- and inter-examiners reproducibility. Once the inter- and intra-examiners agreement was validated with a Kappa score of 0.80, the evaluation of the participants of the study was initiated. The analysis of time-force graph recorded by T-Scan was carried out by a single researcher (principal investigator).

### 2.1. Recordings and Measurements

All participants were assessed for the following: a) patient general information; b) clinical examination to verify the NCCL; c) digital occlusal evaluation by T-Scan; d) questionnaire for oral hygiene habits (Supplementary Materials). Teeth were examined with a sterile mouth mirror and exploratory probe to confirm the presence of NCCL. The Tooth Wear Index (TWI) developed by Smith and Knight [25] was determined to record the three types of teeth structure loss. The TWI index is a qualitative clinical index; it grades the tooth structure loss into five groups, irrespective of the etiology. The criteria for scores are: “0” for no loss of cervical contour; “1” for minimum loss of contour; “2” for cervical defect less than 1mm deep. Defects at the cervical surface ranging from 1 to 2 mm are scored as 3, while 2 mm-deep defects are recorded as 4. The participants in the NCCL group included in the study were scored 2, 3, and 4. Information regarding oral hygiene habits was collected from the participants by a direct face-to-face method. The participants were given clarifications for any doubts regarding the queries, and information was recorded by the investigator. Recorded information included duration, frequency, force, and technique of brushing; the type of toothbrush was also documented. The dietary habits regarding the consumption of citric drinks were also documented. The static occlusal scheme of canine-guided or group function occlusion was examined; occlusal contacts were confirmed with shim stock.

### 2.2. T-scan Analysis

Digital occlusal evaluation was performed with T-Scan analysis (T Scan III, Tekscan Inc, South Boston, MA, USA). The maxillary central incisor diameter was determined to customize the dental arch dimensions. A sensor was placed in the patient’s mouth, with the sensor support position guide between the maxillary central incisors. The sensor handle was held parallel to the occlusal plane, and digital sensors were calibrated with a trial bite to achieve the maximum of three displayed high pink graphics. The mandible was guided to centric relation using the Dawson bimanual method. During closure, initial contact and time to achieve maximum intercuspation (OT) from the initial contact were recorded. The participants were requested to glide the mandible to left and right and perform protrusive movements; the time required from the initial contact to complete disocclusion (DT) was recorded in all excursive mandibular movements. A relative force–time graph was recorded, T-Scan software was utilized to analyze the OT, DT, and force distribution ratios. The occlusal analysis was repeated three times to confirm the outcomes.

### 2.3. Statistical Analysis

Statistical analysis was performed using SPSS 19 (IBM Corp, Armonk, NY, USA). The association of occlusion and other risk factors with the NCCLs was evaluated using Pearson’s correlation and Binary logistical regression. Significance was set to *p* < 0.05.

## 3. Results

**Figure 1:**
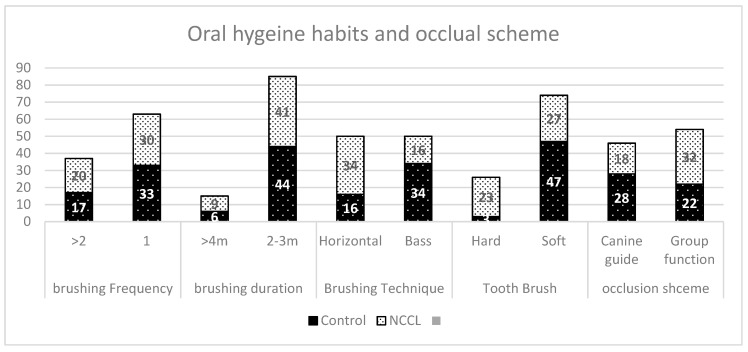
Oral hygiene habits and occlusal scheme in control and non-carious cervical lesion (NCCL) groups.

We found that 68 % of NCCL subjects used the horizontal brushing technique compared to 31% of control subjects (Figure 1). Among the control group, 93% used a soft brush, while 46% of NCCL subjects were used a hard brush. However, no major difference in brushing duration and frequency was observed between control and NCCL groups. An average brushing duration of 2–3 min was recorded in 88% of the control group and 81% of the NCCL group. A brushing frequency over two times was determined for 35% and 40%, of control and NCCL subjects, respectively. The average number of carbonated drink cans consumed in a day by the control group was 1.36, whereas for the NCCL group, it was 1.45.

A higher percentage of participants from the control group (56.3%) had a canine-guided occlusion compared to the NCCL group (31.8%). The multiple linear regression analysis (Table 1) indicated the tooth brush hardness did significantly predict the development of NCCL (β = −0.375, *t*(99) = −3.73, *p* < 0.01).

The T-Scan digital occlusal analysis showed all excursive occlusal contact parameters to be higher in NCCL participants. The mean Right DT, left DT, and protrusive DT were 0.642, 0.686, 0.655, and 1.144, 1.228, 1.06 s for control and NCCL groups, respectively. The mean OT was also longer for NCCL subjects (0.727 s) compared to the control group (0.516 s). The results also showed the NCCL group had initial contact on anterior teeth in 31 participants, and on posterior teeth in 19 participants; in the control group, the majority (32 participants) recorded the initial contact in the posterior teeth. Centralized force was reported in 32 participants of the control group, while only 15 participants of the NCCL group had a centralized force.

On the basis of the results of the study, OT was strongly related to NCCLs, with *r* = 0.455, *p* = 0.001. The other occlusal parameters such as right DT, left DT, and protrusive DT ere also strongly related to NCCL, with the *r*-value of 0.661, 0.642, and 0.534, and *p* = 0.001 (Table 2). The results of binary logistic regression indicated that there was a significant association between OT, right DT, left DT, and tooth brush hardness, with an odd ratio of 70.295, 42.033, 217.327, and 0.045, respectively, and *p* = 0.05 (Table 3). Other parameters like initial contact, protrusive DT, and center of force had no significant association with NCCL.

## 4. Discussion

The uncertain etiology and diagnosis of NCCL have led to a confused approach to its clinical management. Occlusal forces are implicated in the formation of structural defects, while other researchers postulate the role of other factors like saliva, piezo-electric effect, vertical barreling in an acidic environment in the development of the lesions. The purpose of this study was to determine the relation of oral hygiene habits and occlusal parameters in the formation of NCCLs. Earlier studies suggested enamel wear because of horizontal brushing was 2–3 times greater than wear due to vertical brushing [26]. Our results show the use of a harder toothbrush results in increased NCCLs; 93% of the control subjects were reported to use a soft toothbrush compared to 46% of the NCCL subjects. The results are in agreement with the findings of Brandini et al. [27], indicating that the use of medium or hard toothbrushes and a greater force applied during tooth brushing contribute to the development of NCCLs. However, Bizhang et al. [28] reported higher dentin loss with soft bristles than with hard ones. Dyer et al. [29] suggested that soft brushed could lead to increased abrasion, since they incorporate more toothpaste. The toothbrush is just a carrier of the toothpaste, hence, the abrasiveness of the toothpaste could be more significant for tooth structure loss [30]. Recent 3-D image analysis of the abrasive activity of toothpastes showed higher abrasive slurries caused the progression of NCCLs and produced lesions with acute angles [31].

This study demonstrates that the frequency, duration of toothbrushing, and carbonated drink consumption in the NCCL group was higher compared to the control group. Lussi et al. [32] described that the consumption of acidic diets and tooth brushing frequency were associated with increased tooth wear in a six-year clinical study. Abfraction is a wedge-shaped cervical condition; researchers suggest it is the effect of concentrated stress at the cervical region due to non-axial occlusal forces [17].

According to the T-Scan manufacturer’s instruction, the recommended average OT and DT are 0.2 and 0.4 s, respectively [33]. However, Ma et al. [34] showed OT of 0.34 s and DT of 100 s in participants with angles class I occlusion, while Lin et al. [35] reported average OT and DT of 0.47 and 0.89 s, and Haralur et al. [36] described the OT and DT of 0.69 and 0.79 s, respectively, in normal dentate subjects with healthy temporomandibular joints. In this research, NCCL participants presented extended occlusion time and disocclusion time during all mandibular excursive movements. Lee et al. [16] hypothesized that the horizontal occlusal forces originated from mastication, bruxism, and para-functional activity create a fulcrum area in the cervical area. These forces caused stress and torque in the tooth cervical area, leading to the disruption of the thin crystalline structure of enamel [37]. Finite Element Analysis study reported the occlusal forces during inner loading and lateral excursion promoted the concentration of stress in the cervical region. The outer load seemed to be more decisive in affecting the biomechanical behavior of the lower premolars [38]. Hence, a longer excursive lateral DT led to non-axial load for an extended duration. The results of the study are in agreement with the findings of Soares et al. [39], who stated that the oblique load to tooth promoted stress in the cervical region, consequently leading to the initiation and progression of NCCL. The study of Romeed et al. [40] also agrees with the findings of our study; they reported that the maximum stress concentration at the CEJ was generated by lateral loading occlusal forces. Occlusal trauma and group function during lateral excursive movement was recognized as a significant factor for the occurrence of NCCL [41]. The presence of an even small NCCL at the cervical area is predicted to accelerate the progression of the lesion and predispose the teeth to fracture. Hence, dentists are recommended to restore NCCL lesions at an early stage to stop their progression. Composite resin is a restoration material of choice for NCCLs [42]. The management of NCCLs includes the restoration of lost tissue besides the control of all other etiological factors and the rectification of occlusal forces [7].

The limitations of the study include the small sample size and the fact that it examined a single population. Authors recommend longitudinal studies to evaluate the progress of NCCLs due to occlusal interferences. Longitudinal studies are also required to test effective restorative treatments and manage other etiological factors.

## 5. Conclusions

Within the limitations of the study, the following conclusions were drawn. The use of a hard toothbrush and of the horizontal brushing technique were recorded more frequently for the NCCL participants. The mean occlusion time was longer for these subjects compared to the control group. Among the dynamic occlusal parameters, left lateral disocclusion time and right lateral disocclusion time were significantly associated with NCCL.

## Figures and Tables

**Table 1:** Summary regression analysis for oral hygiene habits and occlusal schemes predicting NCCL.

Variable		NCCL	
	B	SE B	β
Brushing frequency	−0.029	0.098	−0.028
Brushing duration	0.015	0.135	0.011
Brushing technique	−0.176	0.100	−0.176
Tooth brush hardness	−0.427	0.115	−0.375 **
Occlusion scheme	0.154	0.093	0.154
*R^2^*	0.266		
F	6.806		

** *p* < 0.01; B: unstandardized beta; SE B: standard error for unstandardized beta; β: standardized beta.

**Table 2:** Pearson correlation coefficients for NCCLs and occlusal parameters.

Groups Score Versus	Control (s)	NCCL (s)	Pearson Correlation	*p* Value
Occlusion time	0.516	0.727	0.455	0.001 **
Rt Disocclusion Time	0.642	1.144	0.661	0.001 **
Lt Disocclusion Time	0.686	1.228	0.642	0.001 **
Pro Disocclusion Time	0.655	1.06	0.534	0.001 **

** Correlation is significant at the *p* ≤ 0.001 level (2-tailed).

**Table 3:** Summary of Binary logistic regression for predicting the NCCL.

Predictor	B	SE B	OR (e^B^)
Initial contact	−1.055	0.833	0.348
Occlusion Time	4.253 *	2.293	70.295
Rt Disocclusion Time	3.738 *	2.197	42.033
Lt Disocclusion Time	5.381 *	2.302	217.327
Pro Disocclusion Time	1.193	1.270	3.296
Centre of Force	1.484	0.957	4.410
Tooth brush hardness	−3.110 *	1.070	0.045

* Correlation is significant at the 0.05 level. B: unstandardized beta; SE B: standard error for the unstandardized beta; e^B^: standardized beta β; OR: odd ratio; Rt: right; Lt: left; Pro: protrusive.

## Supplementary Materials

The following are available online at https://www.mdpi.com/2075-4418/9/2/43/s1. Clinical examination data sheet.

## References

[1] Aw T.C., Lepe X., Johnson G.H., Mancl L. (2002). Characteristics of noncarious cervical lesions: A clinical investigation. J. Am. Dent. Assoc..

[2] Bartlett D.W., Shah P. (2006). A critical review of non-carious cervical (wear) lesions and the role of abfraction, erosion, and abrasion. J. Dent. Res..

[3] Borcic J., Anic I., Urek M.M., Ferreri S. (2004). The prevalence of non-carious cervical lesions in permanent dentition. J. Oral Rehabil..

[4] Bergstrom J., Eliasson S. (1988). Cervical abrasion in relation to toothbrushing and periodontal health. Scand. J. Dent. Res..

[5] Hand J.S., Hunt R.J., Reinhardt J.W. (1986). The prevalence and treatment implications of cervical abrasion in the elderly. Gerodontics.

[6] Oginni A.O., Olusile A.O., Udoye C.I. (2003). Non-carious cervical lesions in a Nigerian population: Abrasion or abfraction?. Int. Dent. J..

[7] Michael J.A., Townsend G.C., Greenwood L.F., Kaidonis J.A. (2009). Abfraction: Separating fact from fiction. Aust. Dent. J..

[8] Nascimento M.M., Gordan V.V., Qvist V., Bader J.D., Rindal D.B., Williams O.D., Gewartowski D., Fellows J.L., Litaker M.S., Gilbert G.H. (2011). Restoration of noncarious tooth defects by dentists in The Dental Practice-Based Research Network. J. Am. Dent. Assoc..

[9] Grippo J.O., Simring M., Coleman T.A. (2012). Abfraction, abrasion, biocorrosion, and the enigma of noncarious cervical lesions: A 20-year perspective. J. Esthet. Restor. Dent..

[10] Nayyer M., Zahid S., Hassan S.H., Mian S.A., Mehmood S., Khan H.A., Kaleem M., Zafar M.S., Khan A.S. (2018). Comparative abrasive wear resistance and surface analysis of dental resin-based materials. Eur. J. Dent..

[11] Ahmed N., Zafar M.S. (2014). Effects of wear on hardness and stiffness of restorative dental materials. Life Sci. J..

[12] Zafar M.S. (2019). Wear behavior of various dental restorative materials. Mater. Technol..

[13] Lussi A., Schlueter N., Rakhmatullina E., Ganss C. (2011). Dental erosion—An overview with emphasis on chemical and histopathological aspects. Caries Res..

[14] Yang J., Cai D., Wang F., He D., Ma L., Jin Y., Que K. (2016). Non-carious cervical lesions (NCCLs) in a random sampling community population and the association of NCCLs with occlusive wear. J. Oral Rehabil..

[15] Rees J.S. (2002). The effect of variation in occlusal loading on the development of abfraction lesions: A finite element study. J. Oral Rehabil..

[16] Lee W.C., Eakle W.S. (1984). Possible role of tensile stress in the etiology of cervical erosive lesions of teeth. J. Prosthet. Dent..

[17] Hammadeh M., Rees J.S. (2001). The erosive susceptibility of cervical versus occlusal enamel. Eur. J. Prosthodont. Restor. Dent..

[18] Litonjua L.A., Bush P.J., Andreana S., Tobias T.S., Cohen R.E. (2004). Effects of occlusal load on cervical lesions. J. Oral Rehabil..

[19] Wiens J.P., Priebe J.W. (2014). Occlusal stability. Dent. Clin. N. Am..

[20] Suit S.R., Gibbs C.H., Benz S.T. (1976). Study of gliding tooth contacts during mastication. J. Periodontol..

[21] Kuroe T., Itoh H., Caputo A.A., Konuma M. (2000). Biomechanics of cervical tooth structure lesions and their restoration. Quintessence Int. (Berl. Ger. 1985).

[22] Dejak B., Mlotkowski A., Romanowicz M. (2005). Finite element analysis of mechanism of cervical lesion formation in simulated molars during mastication and parafunction. J. Prosthet. Dent..

[23] Grenness M.J., Tyas M.J., Osborn J.E. (2009). Mapping a non-carious cervical lesion using stereoimagery and dental casts incorporating optical texture. J. Dent..

[24] Faul F., Erdfelder E., Buchner A., Lang A.G. (2009). Statistical power analyses using G*Power 3.1: Tests for correlation and regression analyses. Behav. Res. Methods.

[25] Smith B.G., Knight J.K. (1984). An index for measuring the wear of teeth. Br. Dent. J..

[26] Mannerberg F. (1960). Appearance of tooth surfaces as observed in shadowed replicas, in various age groups: In long-term studies, after tooth-brushing, in cases of erosion and after exposure to citrus fruit juice. Odontol. Rev..

[27] Brandini D.A., de Sousa A.L., Trevisan C.I., Pinelli L.A., do Couto Santos S.C., Pedrini D., Panzarini S.R. (2011). Noncarious cervical lesions and their association with toothbrushing practices: In vivo evaluation. Oper. Dent..

[28] Bizhang M., Riemer K., Arnold W.H., Domin J., Zimmer S. (2016). Influence of Bristle Stiffness of Manual Toothbrushes on Eroded and Sound Human Dentin—An In Vitro Study. PLoS ONE.

[29] Dyer D., Addy M., Newcombe R.G. (2000). Studies in vitro of abrasion by different manual toothbrush heads and a standard toothpaste. J. Clin. Periodontol..

[30] Litonjua L.A., Andreana S., Bush P.J., Tobias T.S., Cohen R.E. (2004). Wedged cervical lesions produced by toothbrushing. Am. J. Dent..

[31] Sabrah A.H., Turssi C.P., Lippert F., Eckert G.J., Kelly A.B., Hara A.T. (2018). 3D-Image analysis of the impact of toothpaste abrasivity on the progression of simulated non-carious cervical lesions. J. Dent..

[32] Lussi A., Schaffner M. (2000). Progression of and risk factors for dental erosion and wedge-shaped defects over a 6-year period. Caries Res..

[33] Kerstein R.B., Grundset K. (2001). Obtaining measurable bilateral simultaneous occlusal contacts with computer-analyzed and guided occlusal adjustments. Quintessence Int..

[34] Ma F.F., Hu X.L., Li J.H., Lin Y. (2013). Normal occlusion study: Using T-Scan III occlusal analysis system. Zhonghua Kou Qiang Yi Xue Za Zhi.

[35] Lin P.T., Jiao Y., Zhao S.J., Wang F., Li L., Yu F., Tian M., Yu H.H., Chen J.H. (2017). Occlusion and Disocclusion Time Changes in Single Unit Crowns Designed by Functional Generated Path Technique: A Randomised Clinical Trial. Sci. Rep..

[36] Haralur S.B. (2013). Digital Evaluation of Functional Occlusion Parameters and their Association with Temporomandibular Disorders. J. Clin. Diagn. Res. JCDR.

[37] De Las Casas E.B., Cornacchia T.P., Gouvea P.H., Cimini C.A. (2003). Abfraction and anisotropy—Effects of prism orientation on stress distribution. Comput. Methods Biomech. Biomed. Eng..

[38] Zeola L.F., Pereira F.A., Machado A.C., Reis B.R., Kaidonis J., Xie Z., Townsend G.C., Ranjitkar S., Soares P.V. (2016). Effects of non-carious cervical lesion size, occlusal loading and restoration on biomechanical behaviour of premolar teeth. Aust. Dent. J..

[39] Soares P.V., Souza L.V., Verissimo C., Zeola L.F., Pereira A.G., Santos-Filho P.C., Fernandes-Neto A.J. (2014). Effect of root morphology on biomechanical behaviour of premolars associated with abfraction lesions and different loading types. J. Oral Rehabil..

[40] Romeed S.A., Malik R., Dunne S.M. (2012). Stress analysis of occlusal forces in canine teeth and their role in the development of non-carious cervical lesions: Abfraction. Int. J. Dent..

[41] Brandini D.A., Trevisan C.L., Panzarini S.R., Pedrini D. (2012). Clinical evaluation of the association between noncarious cervical lesions and occlusal forces. J. Prosthet. Dent..

[42] Senawongse P., Pongprueksa P., Tagami J. (2010). The effect of the elastic modulus of low-viscosity resins on the microleakage of Class V resin composite restorations under occlusal loading. Dent. Mater. J..

