# Supplementary material for: Association of Non-Carious Cervical Lesions with Oral Hygiene Habits and Dynamic Occlusal Parameters
Source: Diagnostics (Basel). 2019 Apr 12;9(2):43. doi: 10.3390/diagnostics9020043 (PMC6627150; doi:10.3390/diagnostics9020043)
Supplement: Supplementary file 1 [file diagnostics-09-00043-s001.pdf]

Clinical examination data sheet

Patient Serial no: Group: A/B

Age: Gender: F/M

Occupation:

Factors	High	Normal
How Frequently do you brush your teeth?	>2	1
How long do your brush your teeth?	>4 min	2–3 min
Technique of brushing your teeth?	Horizontal	Bass
What type of tooth brush do you use?	Hard	Medium/ Soft
How often you consume citrus fruits /drinks /carbonated drinks		

Type of occlusal scheme

Occlusal scheme	Canine guided	Group function
